# The P38MAPK/ATF2 signaling pathway is involved in PND in mice

**DOI:** 10.1007/s00221-023-06730-6

**Published:** 2023-11-16

**Authors:** Mengjiao Zhu, Si Long, Yizhi Tao, Zhifa Zhang, Zhiqiang Zhou, Xueren Wang, Wei Chen

**Affiliations:** 1 Department of Anesthesiology, The Central Hospital of Wuhan, Tongji Medical College of Huazhong University of Science and Technology, Nanjing Road, Wuhan, 430030 Hubei Province China; 2 Department of Anesthesiology, Tongji Hospital of Tongji Medical College of Huazhong University of Science and Technology, 1095 Jiefang Avenue, Wuhan, 430030 Hubei Province China; 3 Department of Integrated Traditional Chinese and Western Medicine, Zhongnan Hospital of Wuhan University, Wuhan, 430071 Hubei Province China; 4 Department of Anesthesiology, Sun Yat-sen University First Affiliated Hospital, Guangzhou, 510080 Guangdong Province China

**Keywords:** Perioperative neurocognitive disorders, Neuroinflammation, Neuronal apoptosis, Microglial activation, P38MAPK/ATF2 signaling pathway

## Abstract

Accumulating evidence indicates that microglia-mediated neuroinflammation in the hippocampus contributes to the development of perioperative neurocognitive disorder (PND). P38MAPK, a point of convergence for different signaling processes involved in inflammation, can be activated by various stresses. This study aims to investigate the role of the P38MAPK/ATF2 signaling pathway in the development of PND in mice. Aged C57BL/6 mice were subjected to tibial fracture surgery under isoflurane anesthesia to establish a PND animal model. The open field test was used to evaluate the locomotor activity of the mice. Neurocognitive function was assessed with the Morris water maze (MWM) and fear conditioning test (FCT) on postoperative days 1, 3 and 7. The mice exhibited cognitive impairment accompanied by increased expression of proinflammatory factors (IL-1β, TNF-α), proapoptotic molecules (caspase-3, bax) and microglial activation in the hippocampus 1, 3 and 7 days after surgery. Treatment with SB239063 (a P38MAPK inhibitor) decreased the expression of proinflammatory factors, proapoptotic molecules and Iba-1 in the CA1 region of the hippocampus. The number of surviving neurons was significantly increased. Inhibition of the P38MAPK/ATF2 signaling pathway attenuates hippocampal neuroinflammation and neuronal apoptosis in aged mice with PND, thus improving the perioperative cognitive function of the mice.

**Supplementary Information:**

The online version contains supplementary material available at 10.1007/s00221-023-06730-6.

## Introduction

Perioperative neurocognitive disorder (PND), which involves cognitive functional decline to varying degrees, is a common complication after anesthesia and surgery, especially in elderly patients (Evered et al. 2018; Rundshagen 2014). PND affects postoperative recovery and increases the incidence of complications and mortality (Steinmetz et al. 2009). Therefore, elucidating the pathogenesis of PND and identifying treatment strategies will be beneficial for clinical practice (Rengel et al. 2019).

Multiple factors contribute to the development of PND, such as anesthetic drugs, surgical trauma, postoperative pain, and previous cerebrovascular diseases (Rengel et al. 2019). However, the exact mechanisms underlying PND remain unclear, and effective therapeutic strategies are lacking. Neuroinflammation in the hippocampus is responsible for surgery-induced cognitive deficits in aged mice (Cao et al. 2010; Hovens et al. 2014), and microglial activation is a well-known feature of hippocampal neuroinflammation (Feng et al. 2017). Microglia, the resident immune cells in the brain, participate in both the initiation of protective immune responses and the development of invasive inflammation during various CNS disorders (Ransohoff and El Khoury 2015). Upon abnormal stimulation, microglia are gradually activated and secrete numerous inflammatory mediators (IL-1β, TNF-α, Cox-2, etc.), mediating the survival of surrounding cells such as neurons and astroglia, which further exaggerates the inflammatory response, leading to subsequent neuronal apoptosis and irreversible impairment of nervous system function (Block and Hong 2005; Cao et al. 2010). Hence, inhibiting the neuroinflammatory response mediated by activated microglia may alleviate surgery-induced neuronal apoptosis and cognitive dysfunction.

P38 MAPK is a stress-induced kinase and is involved in a wide variety of cellular signal transduction pathways and the phosphorylation of transcription factors and cytosolic proteins, which regulate processes such as cell inflammation and apoptosis (Sui et al. 2014; Wang et al. 2015). Moreover, activated P38 further activates the downstream molecular transcription factor ATF2, which can regulate the expression of target genes and inflammation-related proteins such as TNF-α (Yu et al. 2014a, b). SB239063, a potent inhibitor of the P38 MAPK pathway, ameliorates brain injury and neurological deficits in a rat model of cerebral focal ischemia (Barone et al. 2001; Li et al. 2015). Whether the P38MAPK/ATF2 signaling pathway is involved in hippocampal neuroinflammation mediated by microglia in the context of PND has yet to be elucidated.

Therefore, our current study aimed to prove that activation of the P38MAPK/ATF2 pathway is involved in surgery-induced cognitive impairment in aged mice and that inhibition of the P38MAPK/ATF2 pathway attenuates cognitive disorders by reducing microglia-mediated neuroinflammation and neuronal apoptosis.

## Materials and methods

### Animal protocol

Female C57BL/6 J mice (16 months old, female) weighing approximately 30 g were obtained from the Laboratory Animal Centre of Tongji Medical College, Huazhong University of Science and Technology (Wuhan, China). All mice were acclimated to a new environment for 7 days on a 12:12 h light/dark cycle. The experimental protocols were performed in accordance with the National Institutes of Health (NIH) guidelines for animal care and approved by the Experimental Animal Committee of Tongji Hospital, Tongji Medical College, Huazhong University of Science and Technology.

Ninety mice were randomly divided into three groups: the control (Con) group, surgery (Sur) group and surgery + SB239063 (Sur + SB) group. Mice in the Con group did not receive any special intervention. Mice in the Sur and Sur + SB groups received tibial fracture surgery. Furthermore, mice in the Sur + SB group were administered SB239063 (10 mg/kg, MedChemExpress, USA) orally 60 min before and 6 h after tibial fracture surgery. Mice in all groups were sacrificed on days 1, 3, and 7 after surgery.

### Surgery and anesthesia

After the mice in the Sur and Sur + SB groups were anesthetized with 1.4% isoflurane and 100% oxygen, open tibial fracture surgery with intramedullary fixation was performed. In brief, the right hind limb of mice was shaved and disinfected, and then a midline incision was made on the right hind limb under aseptic conditions. A 0.38 mm pin was inserted into the tibial intramedullary canal, thus achieving intramedullary fixation. The periosteum was then stripped, and the bone was fractured at the midpoint. After surgery, the wound was irrigated, and the skin was sutured with 5–0 Vicryl sutures (Ethicon). To relieve the pain from skin incision, compound lidocaine cream was applied locally to the wound. The mice were then placed in a chamber filled with 1.4% isoflurane and 100% oxygen. The mice were anesthetized for up to 2 h total. Finally, the animals were allowed to recover spontaneously from anesthesia and returned to their own cages, where they were provided ad libitum access to food and water. During the procedure, the body temperature of the mice was maintained at 37 ± 0.5 °C with a heating pad.

### Open field test

Locomotor activity was evaluated by the open field test. A square wooden open field (50 cm × 50 cm × 50 cm) was subdivided into 9 even squares with a white floor. The mice were individually placed in the central zone and allowed to explore for 5 min. The total distance traveled and the number of rearings in the box were recorded with a video tracking system. At the end of each trial, the floor of the chamber was cleaned with 75% ethanol to remove olfactory cues. The open field test was performed one day before the surgery and on days 1, 3, and 7 after surgery.

### Morris water maze test

The MWM was used to evaluate spatial learning and memory in mice. The apparatus consisted of a round pool (120 cm in diameter and 50 cm in height) filled with water that was divided into four quadrants. There was an escape platform in the center of the target quadrant 1 cm beneath the water surface. During the positioning navigation test, the mice were placed in different quadrants of the pool with their heads facing the wall. Each mouse was allowed to search for the platform for 60 s and stay on the platform for 15 s. The time spent searching and mounting the platform was defined as the escape latency, which was used to assess the spatial learning ability of the mice. The mice that failed to find the platform within 60 s were guided manually to the platform and left on the platform for 15 s, and the escape latency was recorded as 60 s. The training was performed four times a day for five successive days before the operation, and the platform was kept in the same location throughout the training process. In the spatial exploration test, we removed the platform, the mice were placed in the quadrant opposite the target quadrant facing the pool wall, and they were allowed to swim freely for 60 s. The time spent searching for the target quadrant (the quadrant where the platform was previously located) and the number of times the mice crossed the original platform position (number of platform crossings) were recorded to assess memory. This test was performed on days 1, 3, and 7. The water in the pool was changed every day and made opaque by nontoxic white paint, and the temperature was maintained at 23–25 °C. The paths of the mice were recorded and analyzed with a computerized video tracking system.

### Fear conditioning test

Fear conditioning was performed to evaluate the hippocampus-dependent learning and memory of the mice. In the training phase, the mice were placed in a conditioning chamber with a stainless steel shock grid floor in the absence of any stimuli for 3 min for adaptation. Then, three pairs of conditioned-unconditioned stimuli were delivered at an interval of 30 s. Each pair of conditioned-unconditioned stimuli consisted of a 30 s, 80 dB tone (conditioned stimulus) and then a 2 s, 0.75 mA electrical foot shock (unconditioned stimulus). In the testing phase, the mice were placed in the original chamber in the absence of stimulation for 5 min. The freezing time of the animals, i.e., the duration for which the animals showed no movement except for respiration, was recorded to assess contextual memory. Cued fear memory was tested by delivering a continuous 3 min tone in the same chamber and recording the freezing time with tracking system software.

### Western blotting

Hippocampal tissues from the mice were homogenized in a mixture of RIPA lysis buffer, PMSF, and phosphatase and protease inhibitors and incubated for 30 min on ice. Then, the lysates were centrifuged at 12,000 rpm for 15 min at 4 °C to remove the sediment. The total protein concentration was determined by a BCA protein assay kit (Boster, Wuhan, China). Subsequently, the samples were mixed with 5 × loading buffer. After denaturation for 10 min at 100 °C, equal amounts of protein (50 μg/lane) from each sample were separated by SDS–PAGE and transferred to PVDF membranes (Millipore, Bedford, MA, USA). The membranes were then blocked with 5% skim milk in TBST for 2 h at room temperature. The blots were incubated overnight at 4 °C with the following primary antibodies: anti-P38 (1:1000, #8690, Cell Signaling Technology, USA), anti-p-P38 (1:1000, #4511, Cell Signaling Technology, USA), anti-ATF2 (1:1000, #35031, Cell Signaling Technology, USA), anti-IL-1β (1:500, #ab200478, Abcam, Cambridge, UK), anti-TNF-α (1:1000, #17590-1-AP, Proteintech, Wuhan, China), anti-caspase 3 (1:1000, #ab44976, Abcam, Cambridge, UK), anti-bax (1:1000, #50599-2-lg, Proteintech, Wuhan, China), anti-bcl-2 (1:1000, #12789-1-AP, Proteintech, Wuhan, China), anti-GAPDH (1:1000, #ab37168, Abcam, Cambridge, UK), and anti-β-actin (1:1000, #A01010, Abbkine). The next day, the membranes were washed 3 times for 10 min each time with TBST and then incubated for 2 h at RT with HRP-conjugated goat anti-rabbit (1:5000, A21020, Abbkine, Carlsbad, CA) or goat anti-mouse (1:5000, A21010, Abbkine, Carlsbad, CA) secondary antibodies. Finally, the blots were detected with a SuperLumia ECL Plus HRP Substrate Kit (K22030; Abbkine, Carlsbad, CA) and photographed by Image Lab software (Bio-Rad, USA).

### Real-time PCR

Total RNA from the hippocampi of mice was extracted. A standard cDNA Synthesis kit (Takara Bio Inc., Japan) was used for real-time PCR. In the 20-μl reaction, 1 μg of total RNA was added. Supplementary Table S1 shows the primer sequences.

The thermal cycling conditions for each real-time PCR included a holding stage at 95 °C for 30 s; 40 cycles of amplification at 95 °C for 5 s and 60 °C for 30 s; and a melting curve stage at 95 °C for 15 s, 60 °C for 1 min, and 95 °C for 15 s. Gene expression was analyzed by 2^−ΔΔct^.

### Immunofluorescence staining

After the behavioral tests, the animals were anesthetized with isoflurane and transcardially perfused with PBS followed by 4% paraformaldehyde (PFA). The brains were rapidly harvested, postfixed in 4% PFA overnight, and then placed in 20% and 30% sucrose at 4 °C until they sank. After being embedded in optimal cutting temperature (20 °C), the brains were cut into 10 µm thick sections by a cryostat microtome and mounted on glass slides. The sections were blocked with 10% donkey serum albumin for 45 min at RT before being incubated with a rabbit anti-Iba-1 antibody (1:200, #ab5076, Abcam, Cambridge, UK) or mouse anti-NeuN antibody (1:100; #ab104224, Abcam, Cambridge, UK) overnight at 4 °C. On the following day, the sections were washed four times for 10 min each time using PBST and then incubated with donkey anti-goat secondary antibody (Abbkine, Carlsbad, CA) for 2 h at room temperature in the dark. Finally, tissue sections were exposed to DAPI for 5 min, and the immunostained sections were visualized with a fluorescence microscope (DM2500, Leica, Germany) equipped with an imaging system.

### Nissl staining

After being anesthetized with 10% chloral hydrate (0.3 ml/100 g) by intraperitoneal injection, the mice were perfused with PBS followed by 4% paraformaldehyde (PFA) via the left ventricle. The brains were removed and kept in PFA at 4 °C. The next day, the brains were embedded in optimal cutting temperature (20 °C) and cut into 8 μm thick sections. The slices were dehydrated in gradient alcohol solutions and then treated with Nissl staining solution for 10 min. The number of Nissl bodies in the hippocampal CA1 region was determined with IPP version 6.0 software to assess neuronal loss.

### Statistical analysis

SPSS version 19.0 was used. All data are expressed as the mean ± SEM. We applied one-way ANOVA followed by post hoc tests to evaluate multiple comparisons. When the data follows a normal distribution, we employ the statistical method of Fisher’s Least Significant Difference (LSD); when the data does not follow a normal distribution, we use Dunnett’s Test. *P* < 0.05 was considered to indicate a statistically significant difference.

## Results

### Hippocampus-dependent memory was impaired in aged mice after anesthesia and surgery

**Fig. 1:**
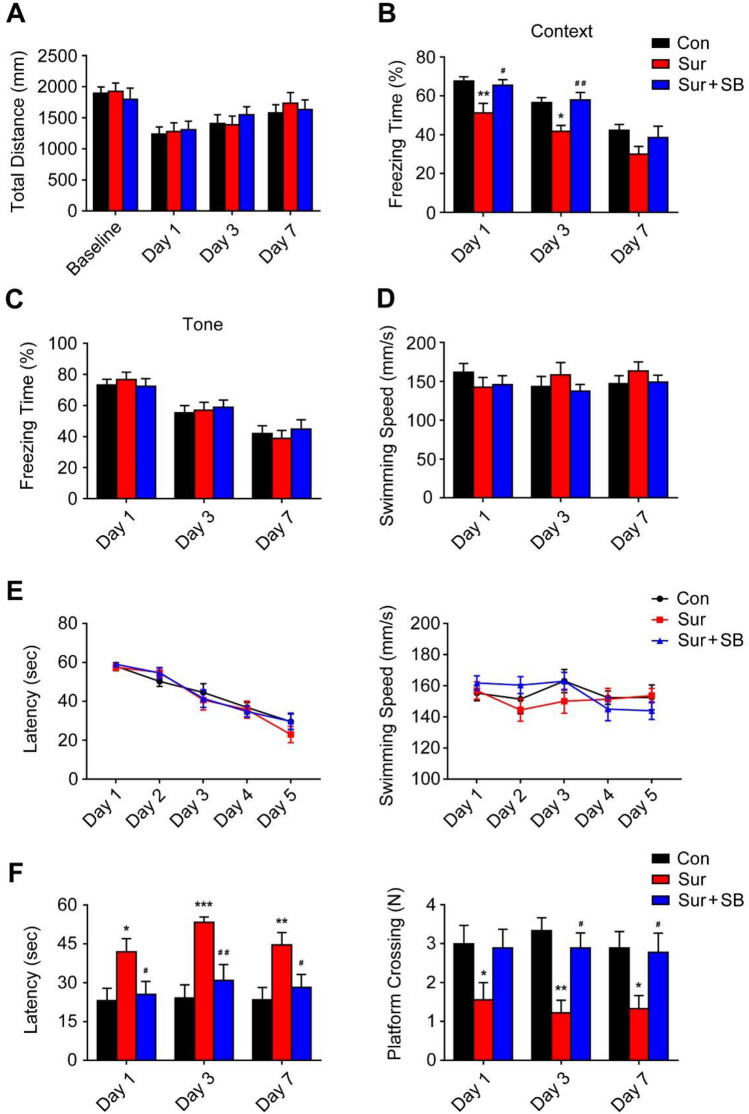
Behavioral tests in aged mice. The open-field test was performed to assess the locomotor activity of the mice by total distance traveled (**A**). The percent freezing time in the context test (**B**) and tone test (**C**). Swimming speed in the MWM on postoperative days 1, 3, and 7 (**D**). The escape latency and swimming speed during training in the MWM before surgery are shown (**E**). Effects of surgery and SB239063 on the escape latency and platform crossings of mice in the MWM (**F**). The data are plotted as the mean ± SEM (*n* = 10). *P < 0.05, **P < 0.01, ***P < 0.001 versus the Con group, *#P* < 0.05, *##P* < 0.01 versus the Sur group

Locomotor activity was assessed by the total distance traveled in the open field chamber. There were no significant differences among the groups (Fig. 1A), indicating that locomotor activity was not affected by surgery or treatment.

We assessed the cognitive functions of mice by the MWM and FCT. In the MWM test, compared with the Con group, the mice with PND in the Sur group had a significantly longer escape latency and made fewer platform crossings on days 1, 3 and 7 (Fig. 1F). However, the mice in the Sur + SB group had a significantly shorter escape latency and made more platform crossings than those in the Sur group (Fig. 1F). In swimming speed, there was no difference among the groups at any time point (Fig. 1D). In the FCT, surgery and anesthesia decreased the freezing time of the mice in the Sur group on days 1, 3 and 7 in the contextual memory test. The freezing time in the contextual memory test was increased in the Sur + SB group compared with the Sur group on days 1 and 3 (*P* < 0.05, Fig. 1B). No significant difference in freezing time in the tone test was observed among the groups (Fig. 1C). These results indicated that anesthesia and surgery can impair the cognitive functions of aged mice.

### The P38MAPK/ATF2 signaling pathway was activated in the hippocampus of aged mice with PND

**Fig. 2:**
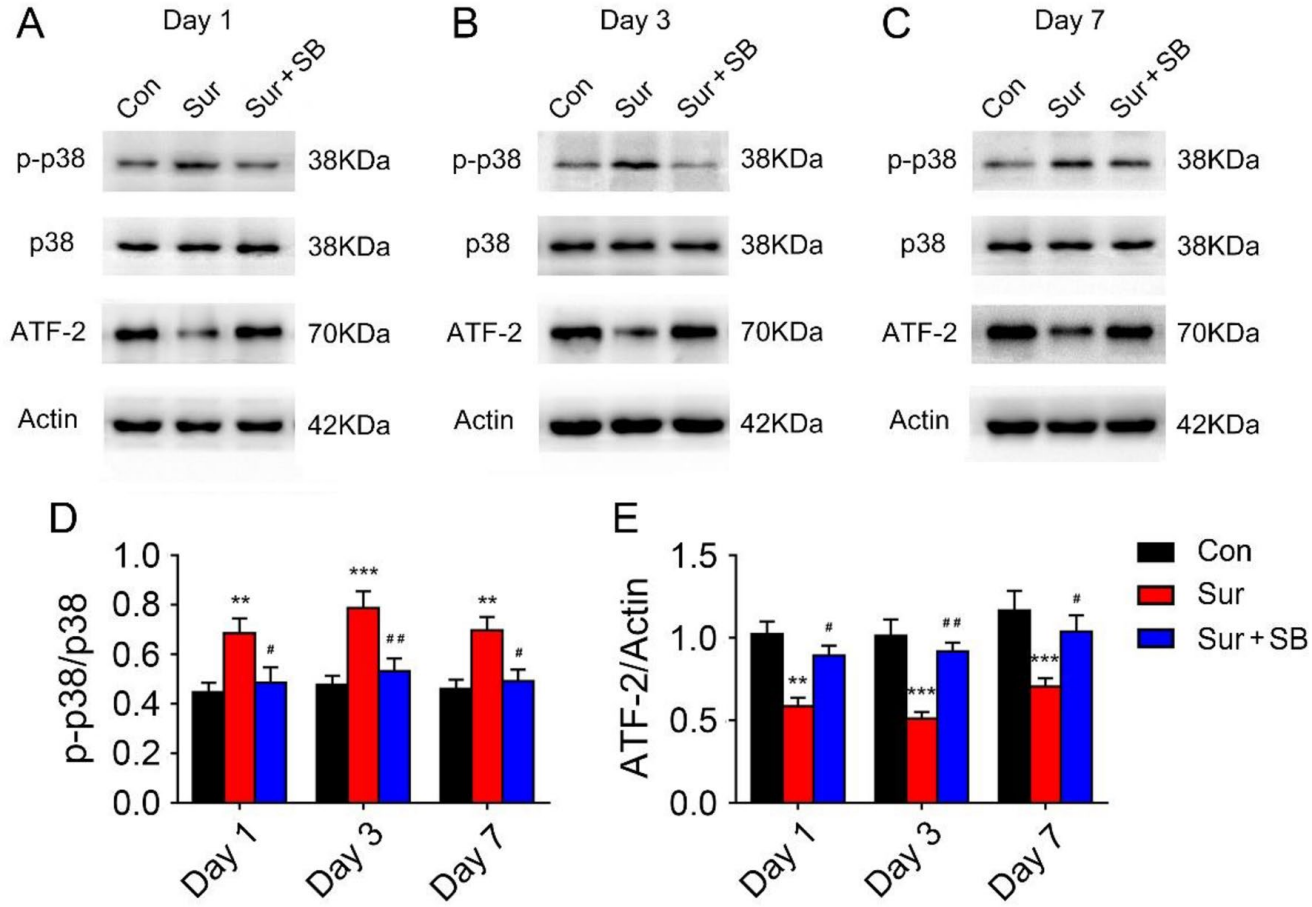
Effects of surgery and SB239063 on the P38MAPK/ATF2 signaling pathway in aged mice on postoperative days 1, 3 and 7. Western blotting showed the expression of P-38, p-P38 and ATF2 in the hippocampus (**A**–**C**). The expression of P-38, p-P38 and ATF2 was normalized to that of GAPDH as an internal control (**D**, **E**). The data are plotted as the mean ± SEM (*n* = 5). **P < 0.01, ***P < 0.001 versus the Con group, *#P* < 0.05, *##P* < 0.01 versus the Sur group

The protein expression of P38, p-P38, ATF2, and p-ATF2 was evaluated by Western blotting (Fig. 2A–C). Rapid phosphorylation of P38 and a decrease in the expression of the downstream factor ATF2 in the hippocampus were observed in mice with PND in the Sur group, but similar alterations were not observed in the Con group (Fig. 2D, E). SB239063, a specific P38 inhibitor, prevented the activation of P38 and restored ATF2 expression (Fig. 2D, E).

The above results showed that activation of the P38MAPK/ATF2 signaling pathway was involved in PND and that SB239063 alleviated cognitive impairment in aged mice by inhibiting the P38MAPK/ATF2 signaling pathway.

### SB239063 reduced the expression of TNF-α and IL-1β in the hippocampus by inhibiting the P38MAPK signaling pathway

We assessed the effects of surgery and SB239063 on the expression levels of the proinflammatory factors TNF-α and IL-1β in the hippocampus by RT–PCR and Western blotting.

**Fig. 3:**
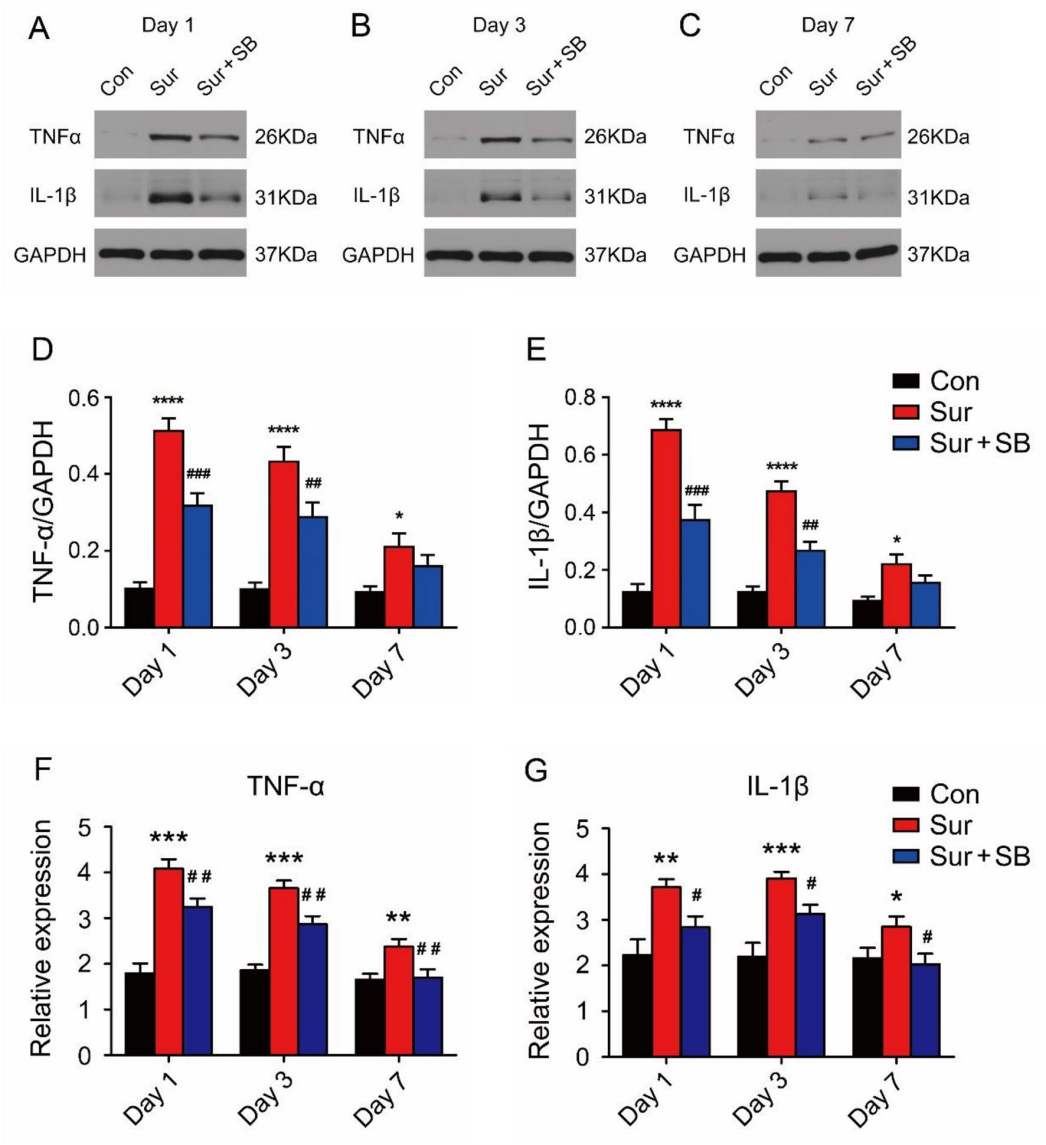
Effects of surgery and SB239063 on proinflammatory factors in the hippocampus on postoperative days 1, 3 and 7. Western blotting showed the protein expression levels of IL-1β and TNF-α in the hippocampus (**A**–**C**). The expression of IL-1β and TNF-α was normalized to that of GAPDH as an internal control (**D**, **E**). Real-time PCR was performed to analyze the relative mRNA expression levels of IL-1β and TNF-α in the hippocampus (**F**, **G**). The data are plotted as the mean ± SEM (*n* = 5). *P < 0.05, **P < 0.01, ***P < 0.001, ****P < 0.0001 versus the Con group, *#P* < 0.05, *##P* < 0.01, *###P* < 0.001 versus the Sur group

Compared with those in the Con group, the TNF-α and IL-1β levels in the hippocampi of the mice in the Sur group were markedly increased on days 1, 3 and 7 (*P* < 0.05, Fig. 3). Mice that received SB239063 treatment exhibited decreased hippocampal protein expression of TNF-α and IL-1β on days 1 and 3 (Fig. 3D, E) and decreased hippocampal gene expression levels of TNF-α and IL-1β on days 1, 3 and 7 (Fig. 3F, G), suggesting that SB239063 could inhibit the production of proinflammatory factors such as TNFα and IL-1β in the hippocampus of mice with PND and that the P38MAPK signaling pathway was involved in this process.

### SB239063 inhibited microglial activation in the CA1 region of the hippocampus

**Fig. 4:**
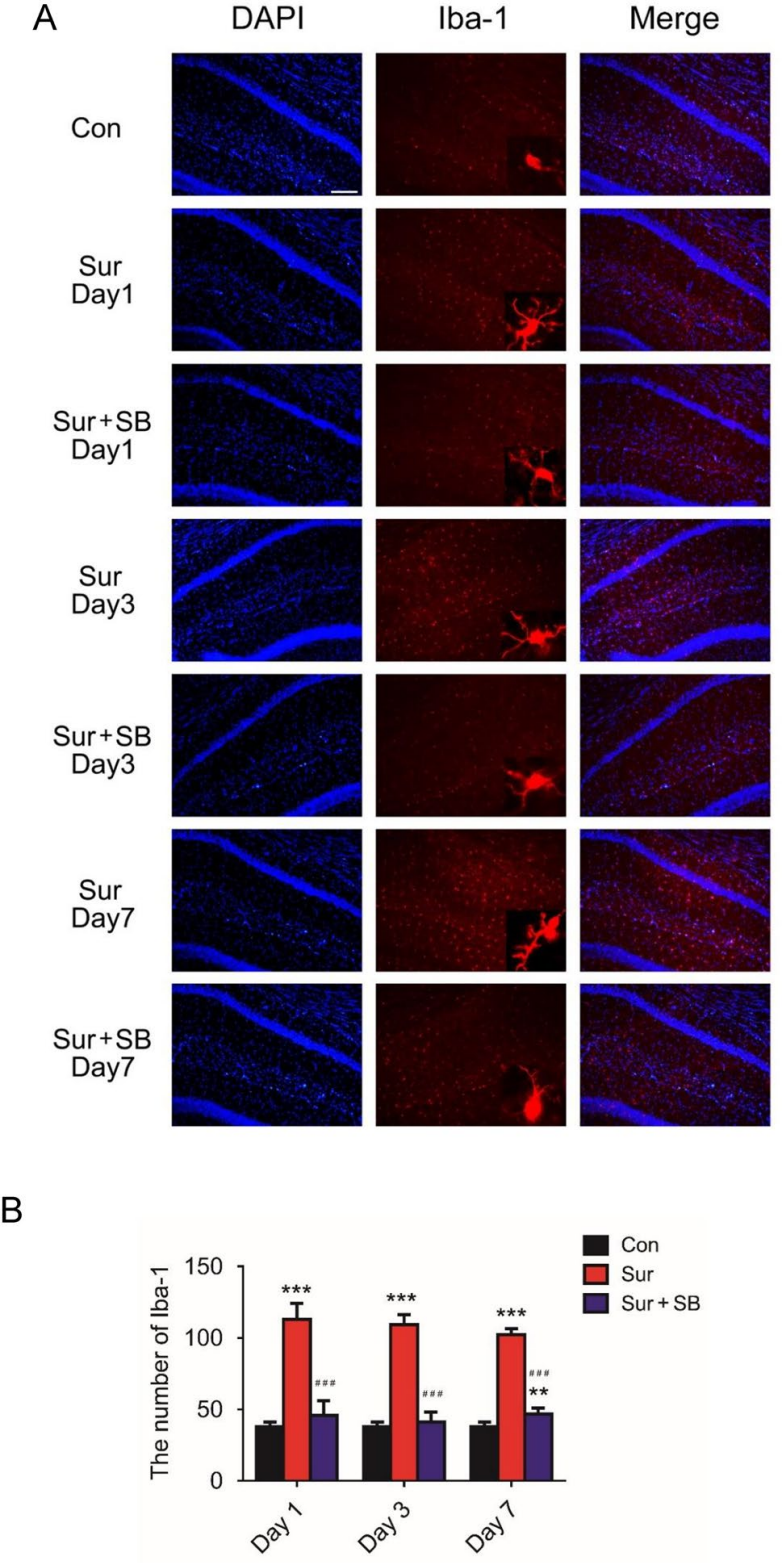
Effect of surgery and SB239063 on microglia in the hippocampal CA1 area of the mice on days 1, 3 and 7. Immunofluorescence images show the number and morphology of Iba-1 (red pixels) in the hippocampus (**A**). Quantitative analyses of Iba-1 (**B**). The data are plotted as the mean ± SEM (*n* = 3). **P < 0.01, ***P < 0.001 versus the Con group, *###P* < 0.001 versus the Sur group. Magnification: 100 × or 400 × . Scale bar = 100 μm

Activated microglia can secrete excessive proinflammatory mediators, which contribute to the development of neuroinflammation. To explore the effect of SB239063 on microglial activation, we used immunostaining to detect the microglial marker Iba-1. Microglial activation in the CA1 region of the hippocampus was markedly increased in the Sur group compared with the Con group on days 1, 3, and 7, as indicated by many Iba1 cells. Activated microglia had larger cell bodies, were poorly ramified, and had short and thick processes (Fig. 4). Nevertheless, these changes were attenuated by SB239063 treatment through suppression of the P38MAPK/ATF2 pathway.

### SB239063 decreased neuronal apoptosis following surgery

**Fig. 5:**
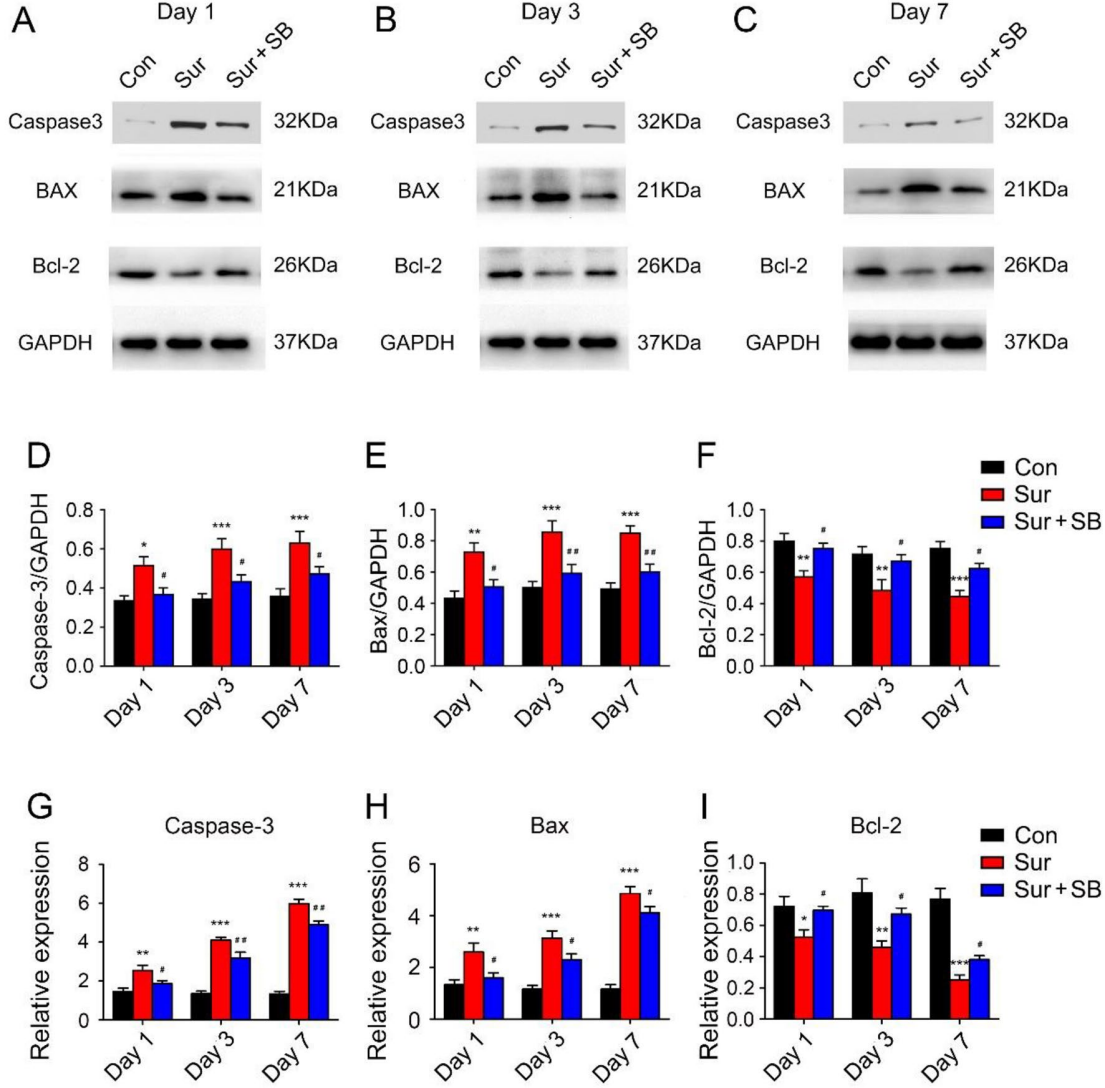
Effects of SB239063 on apoptosis-related proteins in the hippocampus on days 1, 3 and 7. Western blotting showed the expression of bax, bcl-2, and caspase-3 in the hippocampus (**A**–**C**). The expression of bax, bcl-2 and caspase-3 was normalized to that of GAPDH as an internal control (**D**–**F**). Real-time PCR was performed to analyze the relative mRNA expression levels of bax, bcl-2, and caspase-3 in the hippocampus (**G**–**I**). The data are plotted as the mean ± SEM (*n* = 4). *P < 0.05, **P < 0.01, ***P < 0.001 versus the Con group, *#P* < 0.05, *##P* < 0.01 versus the Sur group

**Fig. 6:**
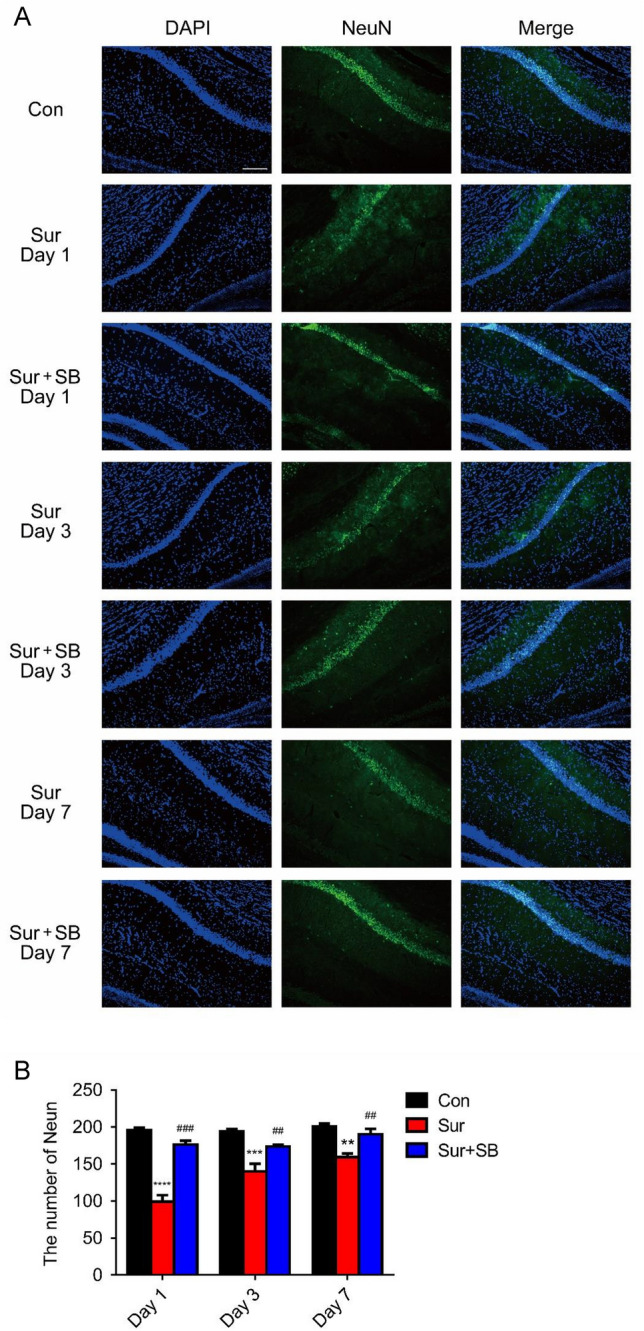
Effect of SB239063 on neurons in the hippocampal CA1 area of mice. Immunofluorescence images show the number of NeuN (green pixels) in the hippocampus (**A**). Quantitative analyses of NeuN (**B**). The data are plotted as the mean ± SEM (*n* = 5). **P < 0.01, ***P < 0.001, ****P < 0.0001 versus the Con group, *##P* < 0.01, *###P* < 0.001 versus the Sur group. Magnification: 100 × . Scale bar = 100 μm

To further confirm the mechanisms by which the P38MAPK/ATF2 pathway contributes to cognitive dysfunction, we assessed neuronal apoptosis by performing immunofluorescence for the neuronal marker NeuN and measuring the expression levels of caspase-3 and Bax/Bcl-2 in the hippocampus. Surgery induced a dramatic increase in caspase-3 and Bax/Bcl-2 expression in the hippocampus and a significant reduction in NeuN expression in the CA1 area of the hippocampus on days 1, 3 and 7. However, compared to the Sur group, the mice in the Sur + SB group exhibited an obvious decrease in caspase-3 and Bax/Bcl-2 expression and an increase in NeuN expression. As shown in Fig. 5 and Fig. 6, treatment with SB239063 significantly inhibited neuronal apoptosis and improved neurogenesis on days 1 and 3. These results suggested that SB239063 may help prevent neuronal injury in the hippocampus induced by surgery.

### SB239063 inhibited neuronal loss induced by hippocampal apoptosis

**Fig. 7:**
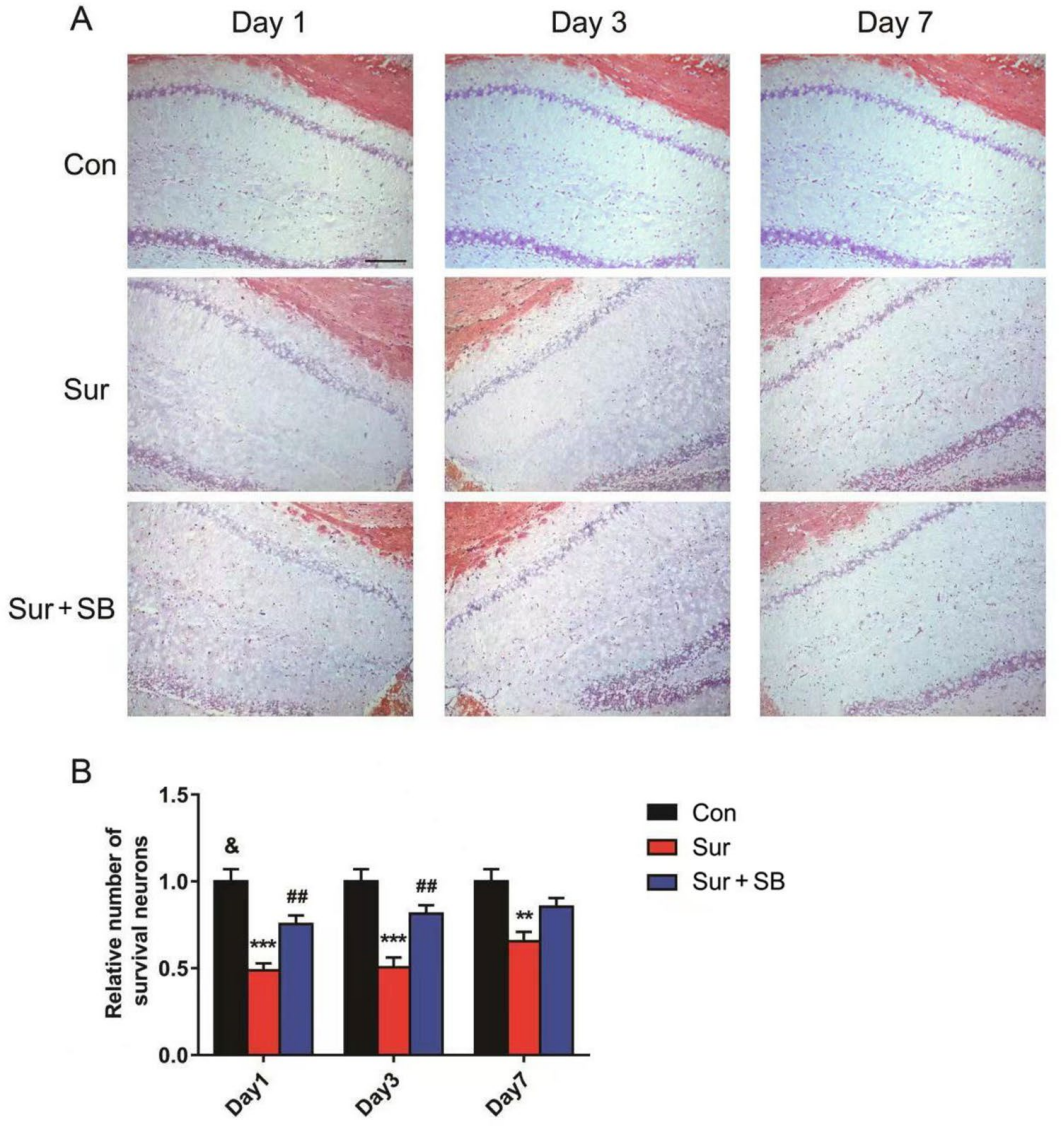
Effect of SB239063 on neuronal loss in the hippocampal CA1 area of mice. Nissl staining reflected the density of surviving neurons in the CA1 of the hippocampus (**A**). Quantitative analyses of Nissl bodies (**B**). The data are plotted as the mean ± SEM (*n* = 5). **P < 0.01, ***P < 0.001 versus the Con group, *##P* < 0.01 versus the Sur group, ^*&*^*P* < 0.05 versus the Sur + SB group. Magnification: 100 × . Scale bar = 100 μm

Nissl staining was conducted to evaluate the number of neurons in the CA1 region of the hippocampus. As shown in Fig. 7, neurons were diffusely deteriorated, and many Nissl bodies were lost in the Sur group compared to the Con group. However, SB239063 increased the number of surviving neurons on days 1 and 3 (*P* < 0.05), but no difference was observed in the number of neurons between the Sur and Sur + SB groups on day 7 (*P* > 0.05). Thus, neuronal loss in the CA1 region was reduced by SB239063.

## Discussion

In the current work, we aimed to study whether the P38MAPK/ATF2 signaling pathway is involved in neuroinflammation, neuronal apoptosis and cognitive impairment in aged mice subjected to orthopedic surgery under isoflurane anesthesia and to explore the neuroprotective mechanisms of SB239063 against PND. Our data suggested that activation of the P38 MAPK/ATF2 signaling pathway in response to surgical trauma plays a critical role in neuroinflammation and cognitive dysfunction. Treatment with SB239063, a P38 inhibitor, prevents PND by ameliorating inflammation and neuronal apoptosis in the hippocampus.

Early clinical studies on PND focused on cardiac surgery, and noncardiac surgery was later found to have similar neurological effects as cardiac surgery (Evered et al. 2011), with the incidence of these effects being higher in elderly patients. According to reports, the incidence of postoperative cognitive impairment in patients over 65 years old was 26% and 10% within weeks and at 3 months after surgery, respectively (van Harten et al. 2012). Twenty-five percent of elderly patients developed PND 7 days after hip replacement (Ji et al. 2013). We used a mouse model of tibial fracture originally described by Harry et al. (Harry et al. 2008) to determine the impact of peripheral trauma on postoperative cognitive function (Xiong et al. 2018). After this surgery, the levels of peripheral and central inflammatory markers in the mice changed significantly, and hippocampal microglia were activated in large numbers. These changes are related to defects in hippocampal neuroplasticity and cognitive function after surgery (Cibelli et al. 2010; Terrando et al. 2010). Clinically, similar changes are observed in elderly people with PND after orthopedic surgery (Hirsch et al. 2016; Neerland et al. 2016). In this study, the Morris water maze and fear conditioning test were used to assess learning and memory, which are important aspects of cognitive function in aged mice. Our results showed that mice with impaired hippocampus-dependent learning and memory exhibited a longer escape latency to reach the platform and fewer original platform crossings in the Morris water maze test and increased freezing time in the contextual fear memory test on Day 1 and Day 3 after surgery.

The hippocampus is an important functional brain area for learning and memory formation. Neuroinflammation of the hippocampus plays a vital role in the progression of cognitive impairment and is probably the critical mechanism underlying PND (Cao et al. 2010; Vacas et al. 2013). Surgical trauma can induce inflammation in the peripheral immune system, causing endothelial cells and phagocytes to secrete and release many inflammatory mediators, such as TNF-α, IL-1β, IL-6, and COX-2 (Cibelli et al. 2010; Terrando et al. 2010). These mediators are not only directly involved in the inflammatory response but also disrupt the permeability of the blood–brain barrier through passive diffusion energy-dependent carriers and other pathways, which can further activate microglia in the nervous system, causing brain injury and cognitive impairment (Wardill et al. 2016; Yu et al. 2014a, b). Furthermore, neuroinflammation may influence neuronal functions either directly or through regulation of intraneuronal pathways, eventually causing neuronal apoptosis, which is another important contributor to the development of PND (Hovens et al. 2014; Li et al. 2017). Excessive neuronal apoptosis can impair the function of the central nervous system and lead to cognitive impairment (Jiang et al. 2018; Peng et al. 2018). Attenuation of the neuroinflammatory response mediated by microglia in the hippocampus of mice can reduce neuronal apoptosis and alleviate cognitive dysfunction (Liu et al. 2019; Zhang et al. 2020). This suggests that hippocampal neuroinflammation and neuronal apoptosis are involved in the occurrence and development of perioperative neurocognitive impairment. In our study, Western blotting and PCR were applied to measure the expression levels of inflammatory and apoptosis-related factors in the hippocampi of aged mice after surgery. Our results showed that the expression levels of proinflammatory and proapoptotic factors were significantly increased, while the expression of antiapoptotic factors was decreased; these changes were associated with impairment of learning and memory in the mice after surgery.

Activated microglia have been shown to be markers of inflammation in the central nervous system and can mediate subsequent neuronal apoptosis. Increasing evidence suggests that activation of microglia is responsible for hippocampal neuroinflammation after surgery and anesthesia (Csuka et al. 2000; Simon et al. 2019). Microglia, which are normally in a resting state, are critical for maintaining homeostasis in the CNS (Ousman and Kubes 2012). Once activated by various stresses, microglia not only secrete much reactive oxygen species and proinflammatory factors to regulate the survival of surrounding cells but also respond to different proinflammatory signals from other cells, leading to a vicious cycle (Lannes et al. 2017; Okuno et al. 2004). Activation of microglia has been proven to be an early sign of neuroinflammation and neuronal apoptosis in neurodegenerative diseases (Lloyd-Burton et al. 2013; Maqbool et al. 2013). Hovens found that activation of hippocampal microglia is closely related to spatial memory disorders (Hovens et al. 2015). In animal models of tibial fractures, activated mast cells in the central nervous system can directly cause hippocampal neuron damage and apoptosis by activating microglia, leading to postoperative cognitive dysfunction (Zhang et al. 2016). Similar to the results presented in our study, surgery led to significant activation of microglia, as indicated by Iba-1 immunoreactivity for up to 7 days. Neuronal apoptosis was observed simultaneously in the CA1 area of the hippocampus. Microglial activation was effectively decreased in the SB group compared with the Sur group, and postoperative neurocognitive disorder was alleviated. Likewise, PCR and Western blotting showed that inhibiting microglial activation prevented the release of proinflammatory cytokines and impaired neuronal function after surgery.

As a mitogen-activated protein kinase (MAPK) family member, p38 MAPK, a point of convergence for different signaling processes involved in inflammation, apoptosis and autophagy, can be preferentially activated by various stresses, such as trauma, proinflammatory cytokines and DNA damage (Duch et al. 2012; Takeda and Ichijo 2002). The potential roles of p38 MAPK signaling pathways in neuroinflammation and neuronal apoptosis have attracted a great deal of attention (Munoz et al. 2007; Roy et al. 2015). BFNM can inhibit LPS-induced inflammatory responses through the P38MAPK/ATF2 signaling pathway and exert an anti-inflammatory effect. Blocking the middle cerebral artery significantly increases the activity of P38MAPK and neuronal apoptosis. Inhibition of p38 MAPK and reduction of p-p38 protein expression can effectively decrease neuronal apoptosis and ameliorate cerebral ischemia–reperfusion injury (Li et al. 2015). SB203580, another p38 MAPK inhibitor, inhibited isoflurane-induced inflammation, oxidative stress, and apoptosis (Liu et al. 2020). In addition, activated p38 MAPK can participate in the cellular apoptosis process by regulating the expression of mitochondrial proapoptotic proteins, such as Bcl-2 family members (Guan et al. 2006; Okuno et al. 2004). Caspase 3 is the executor of cell apoptosis. The regulatory effect between p38 MAPK and caspases is bidirectional, which means that p38 activates caspases to mediate apoptosis, and activated caspases in turn lead to p38 phosphorylation (Moosavi et al. 2007; Song et al. 2011). We detected a significantly higher rate of p38 phosphorylation in the hippocampi of mice with PND, while SB239063 treatment greatly reduced the ratio of p-p38. SB239063 reduced the production of TNF-α, IL-1β, bax/bcl-2, and caspase-3 and the expression level of Iba1 in the hippocampus by inhibiting the p38 MAPK/ATF2 pathway.

The p38 MAPK pathway exerts a wide variety of cellular functions, mostly by regulating multiple downstream molecules. One of the downstream targets of p38 MAPK, ATF2, is expressed ubiquitously, with the highest expression level being found in the brain (Gozdecka and Breitwieser 2012; Hu et al. 1999). This transcription factor is reported to be a component of the basic cell machinery required for neuronal physiology, which may contribute to the complexity of the effects of the p38 MAPK/ATF2 signaling pathway on neuronal inflammation and apoptosis (Pearson et al. 2005; Yu et al. 2014a, b). Once activated, ATF2 transcriptionally regulates various gene targets that control cellular responses, ranging from the transcription factors Jun and Fos to extracellular cytokines and intracellular signaling pathways (Morton et al. 2004). ATF2 knockout mice exhibited impaired migration of some neuronal populations and impaired neurological development (Yu et al. 2014a, b). In Alzheimer’s and Parkinson’s disease, ATF2 expression is downregulated in the hippocampus and caudate nucleus (Pearson et al. 2005), indicating that ATF2 is essential for neuronal viability and normal neurological functions. Rapid and long-lasting suppression of ATF2 expression has been regarded as a common response to neuronal damage in the CNS (Martin-Villalba et al. 1998). Considering that ATF2 is critical for hippocampal neurogenesis and memory processing, we speculated that the expression of ATF2 is responsible for inflammation and neuronal apoptosis in the hippocampus associated with cognitive impairment. Furthermore, our findings confirmed that the upregulation of p-P38 expression and reduction in ATF2 expression were consistent with indicators of inflammation and apoptosis in the hippocampus of aged mice with PND. However, after SB239063 treatment, the expression levels of p-p38 and ATF2 were restored to normal levels, the expression of inflammatory factors was reduced, and excessive activation of microglia and apoptosis of neurons were inhibited, affecting the development of postoperative cognitive dysfunction.

## Conclusions

In summary, our research revealed that activation of the p38MAPK-ATF2 pathway is involved in the development of PND in aged mice. Inhibiting the p38MAPK-ATF2 pathway may be a neuroprotective strategy for cognitive dysfunction induced by surgery through attenuation of microglia-mediated neuroinflammation and inhibition of neuronal apoptosis in the hippocampus.

### Supplementary Information

Below is the link to the electronic supplementary material.

Supplementary file1 (docx 15 kb)

## Data Availability

The data presented in this study are available on request from the first author.
