# Supplementary material for: The P38MAPK/ATF2 signaling pathway is involved in PND in mice
Source: Exp Brain Res. 2023 Nov 16;242(1):109–21. doi: 10.1007/s00221-023-06730-6 (PMC10786957; doi:10.1007/s00221-023-06730-6)
Supplement: Supplementary file 1 — Supplementary file1 (docx 15 kb) [file 221_2023_6730_MOESM1_ESM.docx]

| Gene | Primer | Sequence |
| --- | --- | --- |
| GAPDH | Forward | 5‘-TGAAGGGTGGAGCCAAAAG-3’ |
|  | Reverse | 5‘-AGTCTTCTGGGTGGCAGTGAT-3’ |
| Bax | Forward | 5‘-AGGATGCGTCCACCAAGAAG-3’ |
|  | Reverse | 5‘-GTAGAAGAGGGCAACCACGC-3’ |
| Bcl-2 | Forward | 5‘-GATTGTGGCCTTCTTTGAGTTC-3’ |
|  | Reverse | 5‘-CATATAGTTCCACAAAGGCATCC-3’ |
| Caspase3 | Forward | 5‘-GGAGAAATTCAAAGGACGGG-3’ |
|  | Reverse | 5‘-GCATGGACACAATACACGGG-3’ |
| TNF-α | Forward | 5'-TCCCCAAAGGGATGAGAAGTT-3' |
|  | Reverse | 5'-GAGGAGGTTGACTTTCTCCTGG-3' |
| IL-1β | Forward | 5'-GGGCCTCAAAGGAAAGAATCT-3' |
|  | Reverse | 5'-GAGGTGCTGATGTACCAGTTGG-3' |
| Gene | Primer | Sequence |
| GAPDH | Forward | 5‘-TGAAGGGTGGAGCCAAAAG-3’ |
|  | Reverse | 5‘-AGTCTTCTGGGTGGCAGTGAT-3’ |
| Bax | Forward | 5‘-AGGATGCGTCCACCAAGAAG-3’ |
|  | Reverse | 5‘-GTAGAAGAGGGCAACCACGC-3’ |
| Bcl-2 | Forward | 5‘-GATTGTGGCCTTCTTTGAGTTC-3’ |
|  | Reverse | 5‘-CATATAGTTCCACAAAGGCATCC-3’ |
| Caspase3 | Forward | 5‘-GGAGAAATTCAAAGGACGGG-3’ |
|  | Reverse | 5‘-GCATGGACACAATACACGGG-3’ |
| TNF-α | Forward | 5'-TCCCCAAAGGGATGAGAAGTT-3' |
|  | Reverse | 5'-GAGGAGGTTGACTTTCTCCTGG-3' |
| IL-1β | Forward | 5'-GGGCCTCAAAGGAAAGAATCT-3' |
|  | Reverse | 5'-GAGGTGCTGATGTACCAGTTGG-3' |
